# Neuroprotective Effect of Danggui Shaoyao San via the Mitophagy-Apoptosis Pathway in a Rat Model of Alzheimer's Disease

**DOI:** 10.1155/2021/3995958

**Published:** 2021-09-28

**Authors:** Zhenyan Song, Deyong Luo, Yuke Wang, Yushan Zheng, Peiying Chen, Xiaofang Xia, Chunxiang He, Wenjing Yu, Ping Li, Chen Xiao, Shaowu Cheng

**Affiliations:** ^1^Key Laboratory of Hunan Province for Integrated Traditional Chinese and Western Medicine on Prevention and Treatment of Cardio-Cerebral Diseases, Hunan University of Chinese Medicine, Changsha, Hunan 410208, China; ^2^College of Integrated Traditional Chinese and Western Medicine, Hunan University of Chinese Medicine, Changsha, Hunan 410208, China

## Abstract

Alzheimer's disease (AD) is a serious neurodegenerative disease. While the main pathological characteristic of AD is widely believed to be the accumulation of amyloid-beta (A*β*) in neurons around neurofibrillary plaques, the molecular mechanism of pathological changes is not clear. Traditional Chinese medicine offers many treatments for AD. Among these, Danggui Shaoyao San (DSS) is a classic prescription. In this study, an AD model was established by injecting A*β* 1–42 into the brains of rats, which were then treated with different concentrations of Danggui Shaoyao San (sham operation; model; and Danggui Shaoyao San high-dose, medium-dose, and low-dose intervention groups). The Morris water maze test was used to assess the learning and memory abilities of the animals in each group. Nissl staining was used to detect neurons. Mitophagy was evaluated by transmission electron microscopy and immunofluorescence colocalization. Apoptosis was assessed by terminal deoxynucleotidyl transferase dUTP nick end labeling (TUNEL) assay. The expression levels of autophagy- and apoptosis-related proteins were measured by western blot. Compared to the model group, the groups of AD rats administered medium and high doses of Danggui Shaoyao San showed significantly increased learning and memory abilities (*P* < 0.05), as well as significantly increased autophagosomes in the hippocampus. Moreover, the expression of PTEN-induced kinase 1 (PINK1), Parkin, and microtubule-associated protein light chain 3 (LC3-I/LC3-II) was increased, while that of p62 was significantly decreased (*P* < 0.05). The neuronal apoptosis rate was also significantly decreased, the Bcl-2/Bax ratio was significantly increased, and the cleaved caspase-3 protein expression was significantly decreased (*P* < 0.05). Therefore, Danggui Shaoyao San inhibited neuronal apoptosis in AD rats via a mechanism that may be related to the activation of the PINK1-Parkin-mediated mitophagy signaling pathway.

## 1. Introduction

Alzheimer's disease (AD) is a neurodegenerative disease, the clinical symptoms of which include cognitive decline, declining living ability, and aberrant mental behavior. AD is the most common form of dementia, accounting for 60–80% of cases [1]. The burden of AD on Chinese socioeconomic costs and the global economy is substantial and increasing. In 2015, the expenditures for patients with AD patients in China were US$167.74 billion and are expected to increase to US$2.54 trillion in 2030 [2]. The pathological features of AD are the accumulation of brain *β*-amyloid (A*β*) plaques and A*β* oligomers outside neurons. The main characteristic of AD is the hyperphosphorylation of the tau protein, which causes nerve fiber masses called tau tangles inside neurons, which are diagnostic biomarkers and new targets for drug discovery [3]. A*β* and nerve fiber tangles can affect microglial cells (MGC), induce inflammation, and are related to abnormal glucose metabolism. Inflammatory responses and abnormal glucose metabolism impair mitochondrial function. The accumulation of impaired mitochondria leads to a series of cascade reactions, eventually resulting in neuronal death, which might be the main pathological factors of AD [4]. Mitophagy can identify and eradicate damaged mitochondria to maintain normal mitochondrial function [5].

Danggui Shaoyao San (DSS) was first documented in *Jinkui Yaolue*, written by Zhang Zhongjing in China. DSS is a common prescription for the treatment of AD in traditional Chinese medicine (TCM) and has been used clinically for thousands of years. DSS is also called Dangguijakyak-san or Toki-shakuyaku-san in Japan. In the 1980s, Japanese scholars first reported that DSS improved movement disorders and cognitive function of patients with AD [6]. Clinical and experimental evidence has demonstrated the therapeutic effects of DSS on AD by inhibiting neuronal damage, providing anti-inflammatory and antioxidant activities, enhancing cognitive behavior, increasing energy metabolism, improving mitochondrial damage, and promoting synaptic formation [7–11]. Our previous network pharmacological and transcriptomic analyses found that the therapeutic mechanism of DSS in AD was closely related to mitochondrial dysfunction and mitophagy [12, 13]. Recent research showed that paeoniflorin, an active ingredient of DSS, activated mitophagy to protect SH-SY5Y cells from oxidative damage [14]. The present study injected A*β* oligomers into rat brains to develop an AD model and investigated the neuroprotective effect of DSS on these AD rats based on the mitophagy-apoptosis pathway.

## 2. Materials and Methods

### 2.1. Animals

This study included 60 specific pathogen-free Sprague Dawley (SPF SD) rats weighing 150 ± 20 g (animal production license SCXK [Xiang] 2019-0004). All animals were raised at the SPF Experimental Animal Center of Hunan University of Chinese Medicine (ethical approval number LL-2020071501). All rats were raised separately (three rats per cage) and provided standard rodent feed and water. The light/dark cycle was 12 h.

### 2.2. Drugs and Reagents

DSS medicinal materials including *Angelicae sinensis Radix* (Cat.211054), *Paeoniae Radix Alba* (Cat.211002), *Poria cocos (Schw.) Wolf.* (Cat.212805), *Atractylodes macrocephala Koidz.* (Cat.210133), *Alisma orientale (Sam.) Juz.* (Cat.211203), and *Chuanxiong Rhizoma* (Cat.210712) were purchased from Hunan Sanxiang Chinese herbal medicine Co., Ltd. The production and quality control of medicinal materials were in line with the provisions of Chinese Pharmacopoeia (2015 edition). The composition of DSS was determined by referring to the formula described in the *Synopsis of Prescriptions of the Golden Chamber* by Zhang Zhongjing and combined with modern clinical dosages [7, 8] (*Angelicae sinensis Radix* 9 g, *Paeoniae Radix Alba* 48 g, *Poria cocos (Schw.) Wolf.* 12 g, *Atractylodes macrocephala Koidz.* 12 g, *Alisma orientale (Sam.) Juz.* 24 g, and *Chuanxiong Rhizoma* 24 g). A*β*1-42 was purchased from Thermo Fisher Scientific (USA) (Cat. 03112). The one-step terminal deoxynucleotidyl transferase dUTP nick end labeling (TUNEL) cell apoptosis detection kit (Cat. C1086) was purchased from Shanghai Beyotime Biotechnology Co., Ltd. TOMM20 (Cat. 42406), LC3B (Cat. 43566), and p62 (Cat. 23214) were purchased from Cell Signaling Technology (USA). PTEN-induced kinase 1 (PINK1, cat. DF7742) was purchased from Affinity Biosciences (China). Parkin (cat. Bs-1865R), caspase-3 (cat. Bs-0081R), Bax (cat. Bs127R), Bcl-2 (bs-20352R), and *β*-actin (bs-0061R) were purchased from Beijing Bioss Company (China). Goat anti-rabbit (cat. AP132P), goat anti-mouse (cat. AP124) secondary antibodies were purchased from Sigma-Aldrich (USA). SlowFade Gold Antifade Mountant with 4′,6-diamidino-2-phenylindole (DAPI, cat. S36942); donkey anti-rabbit IgG (H + L) highly cross-adsorbed secondary antibody, Alexa Fluor 488 (cat. A21206); and donkey anti-rabbit IgG (H + L) highly cross-adsorbed secondary antibody, Alexa Fluor 546 (Cat. A10040) were purchased from Invitrogen (USA).

### 2.3. Drug Preparation and Identification

DSS was prepared by water extraction as previously described. The content of the main components was determined for quality control. In brief, the medicinal materials were mixed and made into a powder, soaked in 8-fold (v/w) distilled water for 1.5 h, boiled for 0.5 h, and simmered for 1 h. The filtrate was then collected. The drug residue was then reextracted with 6-fold (v/w) distilled water using the same procedure. The extractions were combined twice and concentrated in a rotary evaporator. Finally, a freeze-dried DSS powder was obtained by vacuum freeze-drying the extract in a lyophilizer for 2-3 days. Approximately, 12.6 g of lyophilized powder was prepared from 100 g of raw materials. Ferulic acid, paeoniflorin, ligustilide, atractylodes lactone I, and alisol B 23-acetate were used as standards to identify the main components of DSS for quality control [13].

### 2.4. Animal Model

In this study, 10 mg A*β*1-42 was dissolved in dimethyl sulfoxide (DMSO) to prepare a 5 mM solution; then, 100 *μ*L of the 5 mM A*β*1-42 was added to phosphate-buffered saline (PBS) to make a 2 *μ*g/*μ*L solution. A*β*1-42 was cultured at 37°C for 7 days. Before injection, the sample was centrifuged at 14000 g for 20 min to remove insoluble oligomers. The supernatant containing the soluble A*β*1-42 oligomer was transferred to a clean tube and stored at 4°C [15, 16]. The rats were fasted for 12 h before the procedure and anesthetized with 0.3% pentobarbital sodium. Next, 5 *μ*L of A*β*1-42 was injected into each side of the cerebral lateral ventricles (intracerebroventricular [ICV] injection) of the rats according to the stereotaxic ATLAS of rat brain (6^th^ edition). The stereotactic coordinates were anterior-posterior (AP), 0.8 mm from the Bregma; medial-lateral (ML), ±1.5 mm from the midline; and dorsal-ventral (DV), 3.7 mm from the skull surface, A*β*1-42 was injected at a rate of 1 *μ*L/min over 5 min, with the needle kept in place 5 min after injection. After the needle was removed, an iodine tincture was applied to the wound and a surgical suture was performed. In the sham operation group, equal doses of physiological saline were injected by ICV, as described above. The animals were kept warm until they regained consciousness and were observed for 24 h. After showing normal activity, they were returned to the SPF laboratory for feeding [17].

### 2.5. Animal Grouping and Treatment Administration

Sixty SD rats were randomly divided by computer into five groups: sham operation, model, DSS intervention low-dose (12 g·kg^−1^ d), DSS intervention medium-dose (24 g·kg^−1^ d), and DSS intervention high-dose (36 g·kg^−1^ d). The dosages were determined based on body surface area conversion between humans and rats, as well as previous studies. The median and high doses were two and four times the adult dose, respectively [13, 18]. The sham operation and model groups were administered an equal volume of normal saline. Two days after the modeling, gavage was performed. According to the above dosages, gavage was administered twice (9:00 a.m. and 6:00 p.m.) for 14 days.

### 2.6. Morris Water Maze

Place navigation: the Morris water maze platform was divided into four quadrants, with a life-saving platform fixed in the first quadrant, 1-2 cm below the surface. During the test, the rats were put into water from four marked sites, respectively, and the escape time was recorded as the time required for the rats to reach the platform and stay for 2 s. If the rats did not find the platform in 1 minute, the rats were guided to the platform for 10 s, and the escape time was recorded as 60 s. This test was carried out twice a day for 5 consecutive days. Space exploration experiment: the rescue platform was removed on the sixth day and the rats were successively lowered from the four quadrants. The times required to shuttle to the platform and staying in the first quadrant for each rat in each quadrant were recorded within 60 s. The Morris water maze video analysis system (ZS-Morris, Beijing Zhongshi Dichuang Technology Co., Ltd.) was used to analyze the water maze trajectory data.

### 2.7. Material Preparation

After the Morris water maze test, the rats were deeply anesthetized with pentobarbital sodium and fixed. A U-shaped incision was made to open the abdominal cavity and the rats were perfused with normal saline at 4°C using a 50 mL syringe. After full perfusion, the brain tissue was placed on ice covered with aluminum foil to separate the left and right brains. The left brain was fixed in a 4% paraformaldehyde solution for pathomorphological examination. The samples examined by transmission electron microscopy were fixed in a glutaraldehyde solution. The right brain was examined by molecular biology, and the isolated hippocampus was placed in a cryopreservation tube in liquid nitrogen for quick freezing.

### 2.8. Transmission Electron Microscopy

Prefixation: the hippocampus was placed in a 3% glutaraldehyde solution precooled at 4°C and incubated for 24 h. Postfixation: the hippocampus was rinsed three times with phosphoric acid buffer and fixed with a 1% osmic acid fixation solution for 2 h. Gradient dehydration, infiltration, and embedding were performed to prepare ultra-thin (70 nm) sections. The sections were dyed with uranium dioxide acetate for 30 min, rinsed with double distilled water 3–5 times, stained with lead citrate for 10 min, and rinsed with double distilled water–3–5 times. After drying, the sections were observed on a transmission electron microscope (Tecnai G2 Spirit TWIN, American FEI Company).

### 2.9. Nissl Staining

The fixed dorsal hippocampus (2.5–4.5 mm from the bregma) was used for coronal sectioning with a pathological paraffin slicer (Finesse E+, Thermo Fisher, USA). The paraffin sections were 3 *μ*m thick. After baking and dewaxing, the samples were dip-stained in 1% toluidine blue at 37°C for 25 min, followed by differentiation in 95% ethanol for 30 s, and gradient dehydration with different concentrations of ethanol; the slides were transparent using xylene, cover glass, and neutral gum mounting.

### 2.10. Immunofluorescent Analysis

After paraffin sectioning, baking, and deparaffinization, a punch was incubated in 0.3% Triton X-100 for 5 min, blocked with 5% normal donkey serum for 1 h, incubated with specific primary antibody overnight, and incubated with translocase of outer mitochondrial membrane 20 (TOMM20) (a mitochondrial membrane-specific marker) antibody at 1 : 200, and LC3B (an autophagy marker) antibody at 1 : 200. The samples were then incubated in fluorescent secondary antibody for 2 h at room temperature, mounted with DAPI-containing mounting tablets, and stored in the dark. A one-step TUNEL cell apoptosis detection kit (green fluorescence) was used for TUNEL staining.

### 2.11. Image Acquisition

Nissl staining was imaged using a microscopic imaging system (McAudi, China), while immunofluorescence was imaged using an A1+ confocal laser microscope (Nikon Company, Japan). Typical images were screened as representative images using suitable laser excitation.

### 2.12. Western Blotting

The rat hippocampal tissue was ground into a powder with liquid nitrogen. The total protein was extracted using the RIPA method and the total protein concentration was determined using the Pierce™ BCA Protein Assay Kit (Cat. 23225, Thermo Fisher Scientific). A total of 30 *μ*g of protein was loaded per well. Sodium dodecyl sulfate-polyacrylamide gel electrophoresis (SDS-PAGE) was performed. The gels were then transferred to a membrane and blocked in 5% skim milk for 1 h before the application of the primary antibodies (PINK1: 1 : 1000, Parkin: 1 : 1000, LC3B: 1 : 1000, p62 : 1:1000, caspase-3 : 1:1000, Bax: 1 : 1000, Bcl-2: 1 : 1000, *β*-actin: 1 : 5000) and incubated overnight at 4°C. The membranes were then incubated with the secondary antibody (1 : 8000) at 37°C in a water bath for 1 h. A Pierce ECL Western kit (cat. 32209, Thermo Fisher Scientific) and Gel Doc XR + gel imaging system (Bio-Rad, USA) were used to image the gels. The relative protein expression levels were calculated using Image-Lab based on the ratio of the target protein to the gray value of *β*-actin.

### 2.13. Statistical Analysis

IBM SPSS Statistics for Windows, version 25.0, was used for statistical analysis. The measurement data were expressed as means ± standard deviation ($\bar{x}\pm s$). *t*-tests and one-way analysis of variance (ANOVA) were used to compare two and more than two groups, respectively. The water maze learning curves were compared by two-way ANOVA. Statistical significance was set at *P* < 0.05. GraphPad 8.0.1 was used to produce the statistical graphs.

## 3. Results

### 3.1. DSS Improves Learning and Memory Impairment in AD Rats

**Figure 1:**
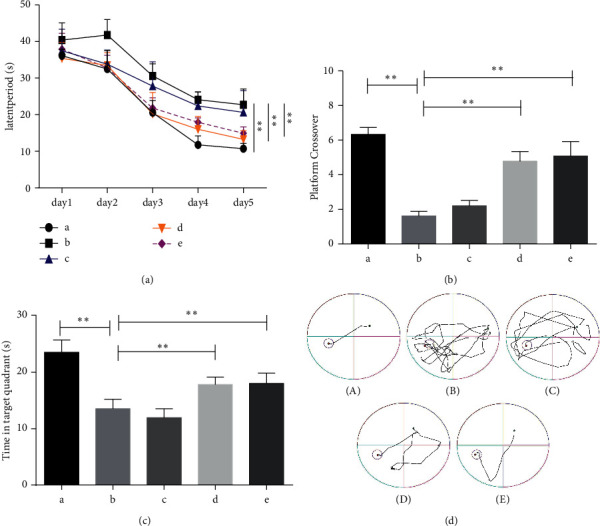
Danggui Shaoyao San effectively improves the learning and memory function of AD rats. A: sham operation group; B: model group; C: DSS low-dose group; D: DSS medium-dose group; E: DSS high-dose group. (a) Escape time in the memory learning phase of the Morris water maze positioning navigation test. (b) Morris water maze space exploration test of the number of crossings on the hidden platform area. (c) Morris water maze space exploration test of the target quadrant stay time. (d) The trajectories of the rats looking for hidden platforms on day 5. *N* = 6 animals/group, ^*∗*^*P* < 0.05, and ^∗∗^*P* < 0.01. AD: Alzheimer's disease; DSS: Danggui Shaoyao San.

A*β*1-42 was injected into both sides of rat ventricles to create the AD model. After developing this model, the drug treatments were administered continuously for 14 days. The results of place navigation in the Morris water maze showed that compared to the sham operation group, AD rats in the model group required a significantly longer time to find a platform (*P* < 0.05) and showed chaotic trajectories. Compared to the model group, the AD rats administered middle and high doses of DSS showed reduced times to find a platform (*P* < 0.05) and a clear movement track (Figures 1(a) and 1(d)). In the space exploration experiment, compared to the sham operation group, the AD rats in the model group showed a significantly reduced number of times reaching the shuttle platform and time spent in the first quadrant (*P* < 0.05). Compared to the model group, the rats administered medium and high doses of DSS showed increased numbers of shuttle platform visits and time spent in the first quadrant (*P* < 0.05) (Figures 1(b) and 1(c)). Thus, DSS improved the learning and memory functions of AD rats.

### 3.2. DSS Protects against Hippocampal Neuron Damage in AD Rats

**Figure 2:**
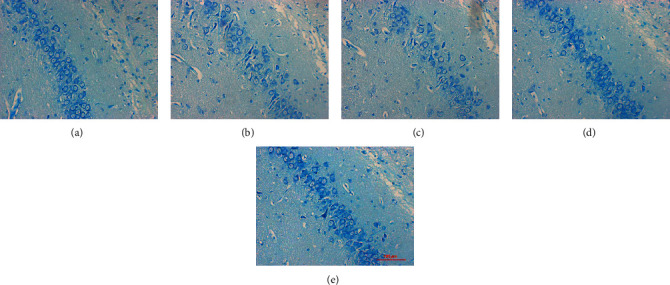
Nissl staining of the hippocampus. (a) Sham operation group. (b) Model group. (c) DSS low-dose group. (d) DSS medium-dose group. (e) DSS high-dose group. DSS: Danggui Shaoyao San.

The Nissl body is an important component of protein synthesis in neurons. When the neuron is stimulated, the Nissl body in the cell is significantly reduced. The results of Nissl staining showed that compared to the sham operation group, the AD rats showed a larger nerve cell gap in the hippocampus; a sparse cell arrangement; slight edema of the cells; some pyknotic and necrotic cells; vacuolar lesions, and reduced Nissl bodies. Compared to the model group, the low-dose DSS group showed decreased edema, necrosis, and vacuole-like cells and increased Nissl bodies in the hippocampal neurons of AD rats. The medium- and high-dose DSS groups showed reduced neuronal damage, clearly visible cytoplasm and nucleus, dense arrangement, and significantly increased Nissl bodies in the hippocampal neurons of AD rats (Figure 2). Thus, DSS had a protective effect on neuronal damage in the hippocampus of AD rats.

### 3.3. DSS Promotes Mitophagy in Hippocampal Neurons of AD Rats

**Figure 3:**
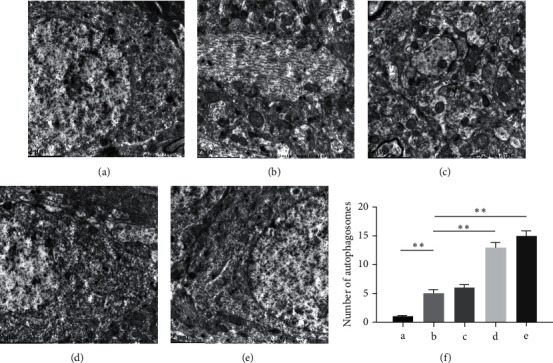
Danggui Shaoyao San promotes mitophagy in hippocampal neurons of AD rats by transmission electron microscope. Formation and distribution of autophagosomes in rat hippocampus. The red arrow indicates the autophagosome. (a) Sham operation group. (b) Model group. (c) DSS low-dose group. (d) DSS medium-dose group. (e) DSS high-dose group. (f) Statistics of the number of autophagosomes. *N* = 3 animals/group; five fields were selected from each group to calculate the number of autophagosomes, ^∗∗^*P* < 0.01. AD: Alzheimer's disease; DSS: Danggui Shaoyao San.

**Figure 4:**
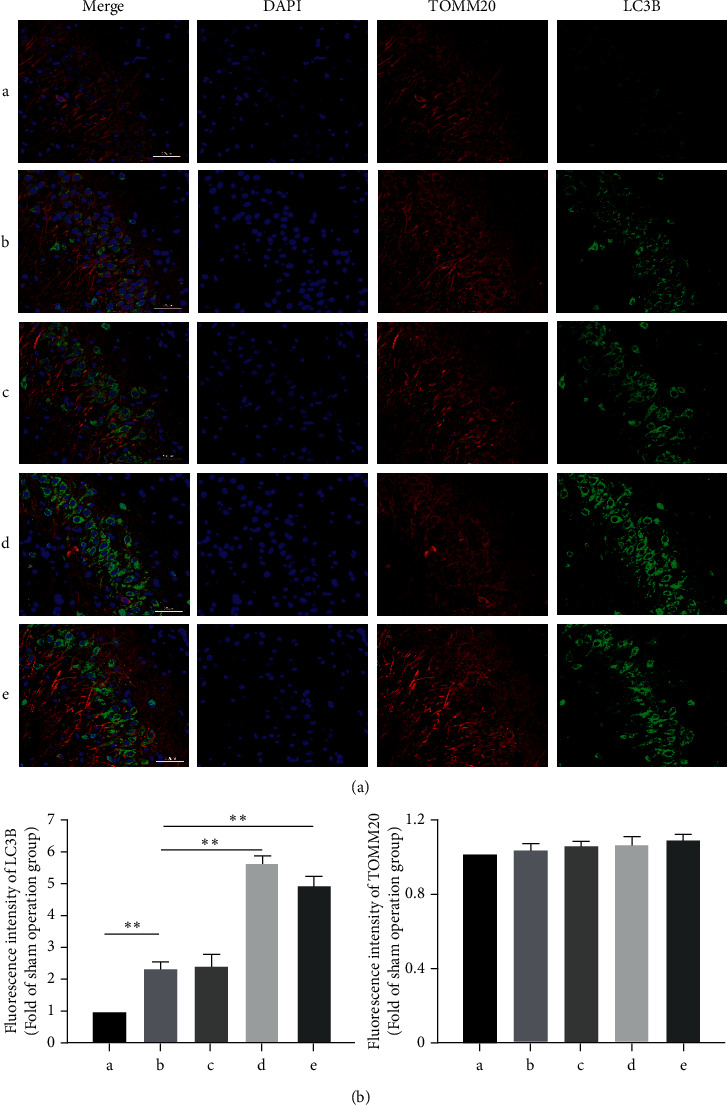
Danggui Shaoyao San promotes mitophagy in hippocampal neurons of AD Rats. A: sham operation group; B: model group; C: DSS low-dose group; D: DSS medium-dose group; E: DSS high-dose group. (a) Immunofluorescence colocalization of mitochondrial membrane TOMM20 (red) and autophagy LC3B (green) markers. (b) Representative images of the fluorescence intensities of LC3B and TOMM20 with or without DSS treatment. *N* = 3 animals/group, ^∗∗^*P* < 0.01. AD: Alzheimer's disease; DSS: Danggui Shaoyao San.

Neurons are sensitive to energy supply and mitochondrial dysfunction leads to neuronal dysfunction [19]. Neurons damaged mitochondria through the autophagy-lysosomal pathway [20]. Mitophagy is one of the main strategies for mitochondrial quality control. We observed mitophagy in the hippocampus of AD rats by transmission electron microscopy. The results showed limited mitophagy in the sham operation group. After A*β* injection, autophagosomes were observed in the cells of the model group. Compared to the model group, the number of autophagosomes increased after DSS treatment. The number of autophagosomes increased significantly after treatment with medium and high doses of DSS (*P* < 0.05). Most autophagosomes had a complete double-membrane or single-membrane structure, while a small portion was dissolved by lysosomes (Figure 3). To further confirm the effect of DSS on mitophagy in hippocampal neurons, we used the mitochondrial membrane marker TOMM20 (red) and autophagy marker LC3 (green) to study the occurrence of mitophagy. Confocal laser scanning showed lower levels of green fluorescence in the cytoplasm of cells from the sham operation group, indicating that there was no obvious mitophagy. After A*β* injection, green fluorescence spots were evenly distributed in the cytoplasm of cells in the model group, and most of the green fluorescence overlapped with the red fluorescence. Compared to the model group, a large number of patchy green phosphors were observed in the cytoplasm after DSS treatment, most of which overlapped with the red fluorescence. Moreover, the number of LC3 positive phosphors increased significantly (*P* < 0.05) (Figure 4).

### 3.4. DSS Promotes Mitophagy by Activating the PINK1-Parkin Pathway

**Figure 5:**
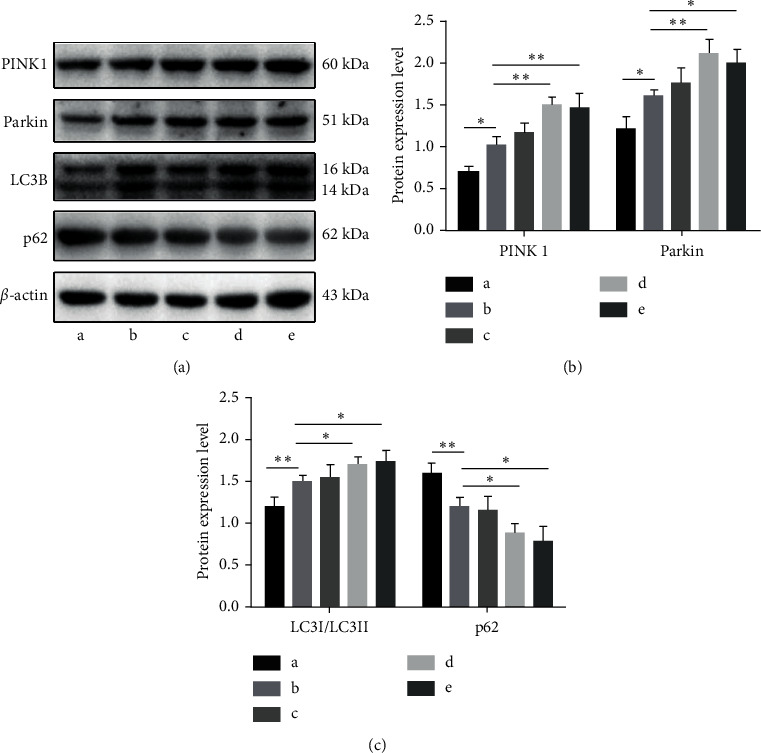
Danggui Shaoyao San activates the PINK1-Parkin pathway to promote mitophagy. A: sham operation group; B: model group; C: DSS low-dose group; D: DSS medium-dose group; E: DSS high-dose group. (a) Detection of PINK1, Parkin, LC3, and p62 protein expression by WB. (b) Statistical results of the relative expression levels of PINK1 and Parkin proteins. (c) Statistical results of the relative expression levels of LC3-I/LC3-II and p62. *N* = 3 animals/group, ^*∗*^*P* < 0.05 and ^∗∗^*P* < 0.01. DSS: Danggui Shaoyao San; WB: western blot.

The PINK1-Parkin signaling pathway is the most typical pathway that mediates mitophagy. This pathway participates in mitophagy degradation together with downstream specific protein signals, including LC3 [21]. The expression levels of PINK1, Parkin, p62, and LC3 in the hippocampus of each group were detected by western blotting. The results (Figure 5) showed that compared to the sham operation group, the expression levels of PINK1, Parkin, and LC3-I/LC3-II in the model group were increased, while the expression levels of p62 were significantly decreased (*P* < 0.05). After treatment with DSS, the expression levels of PINK1, Parkin, and LC3-I/LC3-II were significantly higher than those in the model group, while the expression of p62 was decreased. The difference between the model and DSS groups was statistically significant (*P* < 0.05).

### 3.5. DSS Regulates Apoptosis-Related Proteins and Inhibits Neuronal Apoptosis

**Figure 6:**
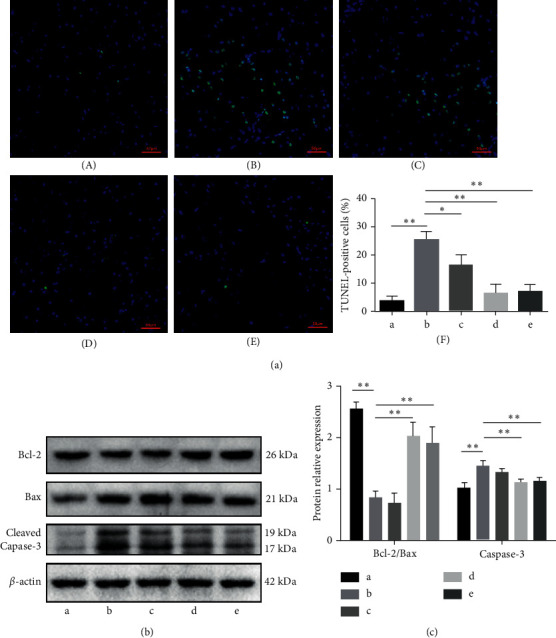
Danggui Shaoyao San inhibits neuronal apoptosis by regulating apoptosis-related proteins. (a) TUNEL-detected neuronal apoptosis. A: sham operation group; B: model group; C: DSS low-dose group; D: DSS medium-dose group; E: DSS high-dose group. F: statistical results of TUNEL-positive cells. (b) Detection of Bcl-1, Bax, and caspase-3 protein expression by WB. (c) Statistical results of the relative expression levels of Bcl-1, Bax, and caspase-3. *N* = 3 animals/group, ^*∗*^*P* < 0.05 and ^∗∗^*P* < 0.01. TUNEL: terminal deoxynucleotidyl transferase dUTP nick end labeling; WB: western blot.

When apoptosis causes DNA fragmentation, the 3′ ends of the DNA fragments usually feature a hydroxyl (-OH) group. After treatment, the tissue sections were labeled with dUTP FITC at the 3′ -OH end of the DNA fragment in the nucleus [22]. The DNA fragments (showing green fluorescence) were detected by fluorescence microscopy using the TUNEL method. The results showed a few green fluorescence points in the sham-operated group, while the group administered A*β*1-42 showed many green fluorescence points. The groups treated with DSS showed a significantly lower number of green fluorescence points compared to that in the model group (Figure 6(a)). Compared to the sham operation group, the apoptosis rate of the rats injected with A*β*1-42 (the model group) was (25.64 ± 4.89)%, significantly higher than that of the sham operation group (3.83 ± 2.94)% (*P* < 0.05). Compared to the model group, the apoptosis rate of the DSS low-dose group was 16.42 ± 6.43%, while that of the DSS medium-dose group was 6.55 ± 5.36%. The apoptosis rate in the DSS high-dose group was 7.12 ± 4.26%, a statistically significant difference (*P* < 0.05) (Figure 6(a)).

Under A*β* stimulation, excessive DNA damage leads to poly ADP-ribose polymerase (PARP) and p38 (Thr 180/Tyr 182P activation), causing increased caspase-3 activation and Bax expression and decreased Bcl-2/Bax ratio, which leads to neuronal apoptosis [23]. The regulatory effect of DSS on Bcl-2/Bax and caspase-3 was detected by western blotting. The results (Figures 6(b) and 6(c)) showed that, compared to the sham operation group, the Bcl-2/Bax ratio was significantly decreased, and cleaved caspase-3 protein expression was significantly increased after A*β* injection (*P* < 0.05). Compared to the model group, the Bcl-2/Bax ratio showed a gradient increase, and the cleaved caspase-3 protein expression showed a gradient decrease after DSS treatment (*P* < 0.05). The differences in the effects of the medium- and high-dose DSS interventions were statistically significant.

## 4. Discussion

At present, the efficacies of the drugs used to clinically delay AD progression (e.g., memantine and donepezil) are not ideal [24]. Natural resources or herbal formulations, which can act on multiple targets simultaneously rather than single compounds focused on a specific mechanism, are a promising approach to the treatment of AD. As one of the most widely used TCM formulations, DSS is marketed as an effective drug for the prevention and treatment of cognitive decline in Japan, South Korea, and China [25, 26]. Pharmacological evidence supports the use of DSS as a potential treatment for AD. The pharmacological effects of DSS include improving memory dysfunction, regulating monoamine neurotransmitter metabolism, increasing superoxide dismutase (SOD) level, and protecting the cortical ultrastructure caused by aging [7]. Some of the active ingredients of DSS have also been studied in the context of improving cognition. JD-30, the active ingredient extracted from DSS, reduces A*β* deposition and ameliorates the induced inhibition of the long-term potentiation of the CA1 region in the rat hippocampus [11]. Ferulic acid, the main organic acid component of *Angelicae Sinensis Radix* and *Chuanxiong Rhizoma*, can directly change the kinetics of A*β* fibril formation, as well as its antioxidant and anti-inflammatory properties, which have demonstrated neuroprotective ability [27]. Paeoniflorin, a pinane monoterpene bitter glycoside isolated from *Paeoniae Radix Alba*, has protective effects against A*β*-induced neurotoxicity by decreasing glutathione levels, inhibiting nitric oxide synthase (NOS) activity and nitric oxide (NO) levels, and decreasing cyclophosphamide (CP) and malondialdehyde (MDA) levels [28]. Ligustilide ameliorates aging-induced memory deficits by regulating mitochondrial function and inhibiting oxidative stress and neuroinflammation in SAMP8 mice [29]. Our research aimed to observe the neuroprotective effect of DSS on an A*β*1-42-induced AD rat model through the regulation of PINK1-Parkin-mediated mitophagy.

A recent study demonstrated the significantly positive effect of DSS treatment on cognition in APP/PS1 mice. As the most common and important clinical indicator of AD, cognitive ability is an important target for the treatment of AD [9]. The results of the water maze test showed impairment of learning and memory in the AD rats compared to those in the normal mice. For instance, the AD model group required the longest average swimming time at the positioning platform. After treatment with DSS, the mean swim time decreased significantly. The Nissl and TUNNEL staining data showed significantly reduced neuronal damage in the hippocampus, reduced neuronal apoptosis, clearly visible nerve cell cytoplasm and nucleus, densely arranged cells, and significantly increased numbers of Nissl bodies in AD rats after DSS treatment. These results suggested that DSS inhibited A*β*-induced neuronal apoptosis and had a protective effect on hippocampal neuronal injury in AD rats.

The pathological characteristics of AD are mainly related to the deposition of A*β* protein and cytopathy caused by neurofibrillary tangles (NFTs). Another prominent feature of AD is mitochondrial dysfunction [30]. A*β* clearance is inhibited as well as its accumulation in neurons in AD. A*β* can cause various mitochondrial dysfunctions, including oxidative stress, mitochondrial DNA damage, decreased cytochrome c oxidase (COX) activity, decreased membrane potential, and overall damage to mitochondrial dynamics [31, 32]. Continuous increase in mitochondrial dysfunction causes decreased mitochondrial biology and ATP production; thus, insufficient energy is produced for synaptic vesicles, leading to synaptic dysfunction [33]. When mitochondria are damaged, the mitochondrial membrane potential decreases and PINK1-Parkin-mediated mitophagy degrades damaged mitochondria [21]. The transgenic mouse model of AD of the human brain showed human amyloid precursor protein (HAPP) and Parkin-mediated mitophagy. During disease development, the level of cytoplasmic Parkin decreases, leading to increased mitochondrial dysfunction [34]. Recent reports suggested an impaired mitophagy pathway as a potential contributing factor in AD. Activation of the Parkin-dependent mitophagy pathway led to increased recruitment of Parkin, LC3, and P62 to damaged mitochondria in mutant hAPP neurons and the brains of patients with AD [34]. These findings suggest that mitophagy is induced at the early stage of disease progression, consistent with our observations of increased mitophagy in AD rat brains. However, with disease progression, there was a significant reduction in cytosolic Parkin levels in samples from patients with AD, indicating inefficient mitophagy [35]. This evidence suggests that mitophagy dysfunction is the main cause of the pathological deterioration of AD disease. Therefore, the promotion of mitophagy might be a feasible target for the exploration of treatment protocols for neurodegenerative disorders. *β*-Asarone has been shown to effectively promote PINK1-Parkin-mediated mitophagy and improve the learning and memory ability of A*β*1-42-induced AD rats [36]. The results of the present study showed increased PINK1 and Parkin expression in the brain of AD rats after DSS intervention, while mitophagy was continuously activated. The electron microscopy and immunofluorescence colocalization findings further suggested significantly increased mitophagy in the brains of AD rats after DSS intervention. In addition, ligustilide, a major component of DSS, improved mitochondrial function by alleviating mitochondrial fusion and fission disorders in SAMP8 mice [29]. DSS may also improve A*β*-induced mitochondrial dysfunction, which may be related to the mitophagy activation and rapid clearance of damaged mitochondria.

Apoptosis follows an external pathway related to a tumor necrosis factor receptor family member or an internal pathway related to the mitochondrial release of cytochrome C [37]. The two pathways are combined at the end of the caspase cascade, in which caspase-3 activation is the last step, triggering protein hydrolysis, cytoskeleton disintegration, and DNA fragmentation [38]. Bax is mainly localized in the cytoplasm. When it contacts the activation signal, it triggers its translocation to mitochondria, enters the outer membrane to form pores, destroys mitochondrial transmembrane potential, and initiates programmed cell death. Bcl-2 can interact with Bax to inhibit its further activation [39]. Under the toxic stimulation by A*β*, the number of proapoptotic Bax proteins also exceeded the number of antiapoptotic Bcl-2 proteins. The mitochondrial membrane integrity also begins to weaken, with increased permeability and polarization. Membrane depolarization and pore opening allow the release of cytochrome C, leading to apoptosis. Therefore, mitophagy pathways must intervene to eliminate dysfunctional mitochondria rather than the whole cell [21]. Previous studies have shown that A*β* accumulation causes mitochondrial dysfunction and neuronal apoptosis in APP/PS1 mice through oxidative stress, which may be caused by Bcl-2 downregulation and caspase-3 upregulation [40]. In addition, paeoniflorin attenuated reactive oxygen species (ROS) production in SH-SY5Y cells and regulated the mitochondrial apoptotic pathway, including the inhibition of Bax/Bcl-2 ratio, cytochrome c release, decreased mitochondrial membrane potential, and decreased caspase-3 and caspase-9 activities [41]. These results were consistent with our findings that DSS inhibited the Bax/Bcl-2 ratio and caspase-3 activity.

The results of this study demonstrated that DSS mitigated A*β*-induced cognitive decline in rats. A 2-week administration of DSS protected learning and memory, promoted mitophagy, and reduced apoptosis. However, the pathogenesis of AD is complex and unclear; moreover, the pharmacological effect of DSS in the treatment of AD cannot be fully elucidated based on the neurological damage induced by A*β* toxicity alone. Multiple animal models or clinical trials are necessary to further confirm the pharmacological effects of DSS in AD. In addition, the complexity of DSS prescription components and the diversity of therapeutic targets make it difficult to fully explain the pharmacological effects based on a single mechanism; thus, studies at the system and omics levels should be considered [42, 43].

In conclusion, A*β* accumulation in neurons in the brain caused mitochondrial dysfunction and neuronal apoptosis. DSS inhibited neuronal apoptosis in AD rats, the mechanism of which may be related to the activation of the PINK1-Parkin-mediated mitophagy signaling pathway to play a neuroprotective role in AD.

## Data Availability

The raw data supporting the conclusions of this manuscript will be made available by the authors, without undue reservation, to any qualified researcher.
